# Thickness-dependent Magnetic and Microwave Resonance Characterization of Combined Stripe Patterned FeCoBSi Films

**DOI:** 10.1186/s11671-018-2506-5

**Published:** 2018-04-12

**Authors:** Li Zhang, Yaoming Liu, Hanyu Zheng, Wenbin Zhu, Min Zhang, Linbo Zhang, Peiheng Zhou, Haiyan Chen, Xin Wang, Haipeng Lu, Jianliang Xie, Longjiang Deng

**Affiliations:** 1 State Key Laboratory of Electronic Thin films and Integrated Devices, National Engineering Research Center of Electromagnetic Radiation Control Materials, Key Laboratory of Multi-spectral Absorbing Materials and Structures of Ministry of Education, Chengdu, 610054 China; 2 School of Electronic Science and Engineering, University of Electronic Science and Technology of China, Chengdu, 610054 China

**Keywords:** Soft magnetic materials, High frequency properties, EMI absorbers

## Abstract

In this paper, we fabricated a series of FeCoBSi multistoried patterned magnetic films with different thickness by traditional UV lithography method and DC sputtering deposition. Broad resonance band phenomenon was observed during high frequency property characterization, with full width half maximum (FWHM) of 4 GHz when the film thickness is 45 nm. The broad resonance band effect was contributed to the existence of multiple resonance peaks due to different stripe width of the combined stripe pattern, which induced distinguish shape anisotropic field in each stripe. Each resonance peak was independent due to the gap between the stripes, leading to a controllable method to tune the microwave properties of such structure. With thickness varied, the resonance band could be altered according to the mathematic prediction. This work presents an effective method for tuning the microwave resonance characterization in magnetization dynamic.

## Background

With the rapid development of telecommunication technology, the problems of electromagnetic inference (EMI), which deteriorate the performance of such systems in high frequency, attract public attentions significantly [1–5]. In order to satisfy the requirements of EMI shielding materials, broadband and controllable resonances of magnetic films are desired [6, 7]. Meanwhile, a high damping factor at designed frequency would make contribution to realize promising EMI devices [8, 9]. Due to in-plane uniaxial anisotropy of a film could lead to well soft magnetic properties at gigahertz frequency, hence, better absorption properties, several methods including induced magnetic field [10], induced stress [11] during deposition, multilayer design [12], and post-annealing under external magnetic field [13, 14], were investigated. Besides, patterned magnetic films with induced shape anisotropy designed by artificial structure draw great public attention due to its controllable and robust properties [15, 16]. In light of this, double-stripe patterned FeCo-based magnetic film were proposed in our former work [17]. Broad resonance band with double resonance peaks phenomenon was observed during experiment, which ascribed to superposition of double resonance source contributed by independent magnetic stripes.

Therefore, in this paper, in order to expand the resonance band furthermore, we introduced a unique combined stripe patterned FeCoBSi thin films containing various stripe with five different widths and analyzed microwave resonance characterization due to multiple resonance peaks with the Landau-Lifshitz-Gilbert (LLG) processional motion formulism. The broad resonance band phenomenon was enhanced with full width half maximum (FWHM) of 4 GHz at thin thickness, i.e., 45 nm for our experiments. Meanwhile, the alteration of resonance frequency could be predicted by the mathematic formula related to demagnetization factors. The results could be further illustrated by the shape-induced effective anisotropy filed due to distinguished stripe width, which made it possible to control by the traditional lithography process in the actual application.

## Experiment

**Fig. 1:**
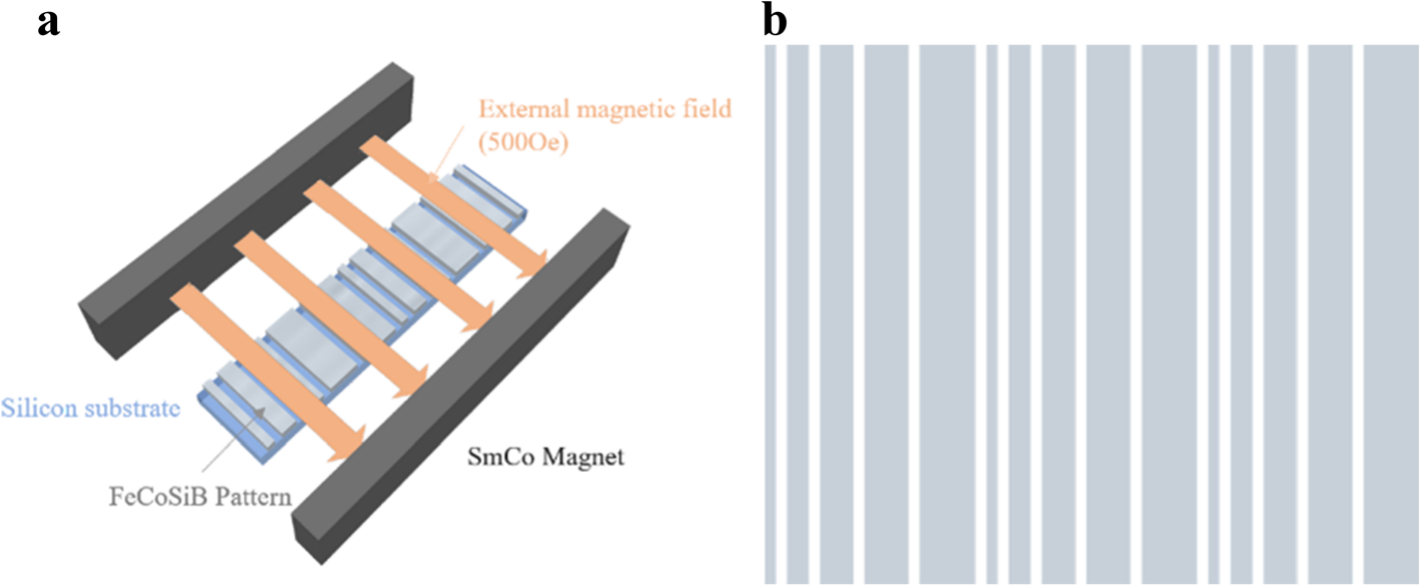
The scheme of the external induced magnetic field during deposition (**a**) and combined stripes patterned magnetic films (**b**). The width of each stripe were 5, 10, 15, 20, and 25 μm, respectively. The width of gap between two stripes was fixed at 5 μm. Lift off process was done after deposition to expose the final structure of film

Fe_66_Co_17_B_16_Si_1_ thin films with different thickness were deposited on silicon (111) substrates by DC magnetron sputtering at room temperature. An external magnetic field of 500 Oe was applied along the short axis of the substrate to induce in-plane uniaxial anisotropy as showed in Fig 1. Traditional ultraviolet (UV) lithography technology and lift-off method were used to fabricate the combined stripe patterns. A combined stripe patterned FeCoBSi films containing various stripe with different width were processed. The stripes were arrange successively with width sequence as 5, 10, 15, 20, and 25 μm respectively. A separation gap of distinctive stripes was fixed at 5 μm. The thickness of the patterned films varied from 45 to 135 nm.

The thickness of the films was determined by the cross-sectional observation with scanning electron microscopy (SEM). Correspondent static properties of magnetic film, i.e., hysteresis loops, were measured by vibrating sample magnetometer (VSM). Microwave properties were characterized by a shorted micro-strip transmission line perturbation method connected to a vector net analyzer in the frequency range of 0.5–6 GHz.

## Results and discussion

Figure 1a shows the scheme of the deposition setup with external induced magnetic field. A 500 Oe external magnetic field was applied during deposition in order to induce in-plane uniaxial anisotropy. Lift-off method was processed after deposition to expose to patterned structure of films. Figure 1b exhibits the combined stripe patterned structure of our magnetic films. The sequence of width for each stripe corresponds to 5, 10, 15, 20, 25 μm, respectively, while the gap between each stripe was fixed at 5 μm. According to our previous work, there were no obvious crystalline peaks except Si (111) from the substrates during XRD measurement [18]. Hence, the crystalline structure of our films was amorphous or nanocrystalline.

**Fig. 2:**
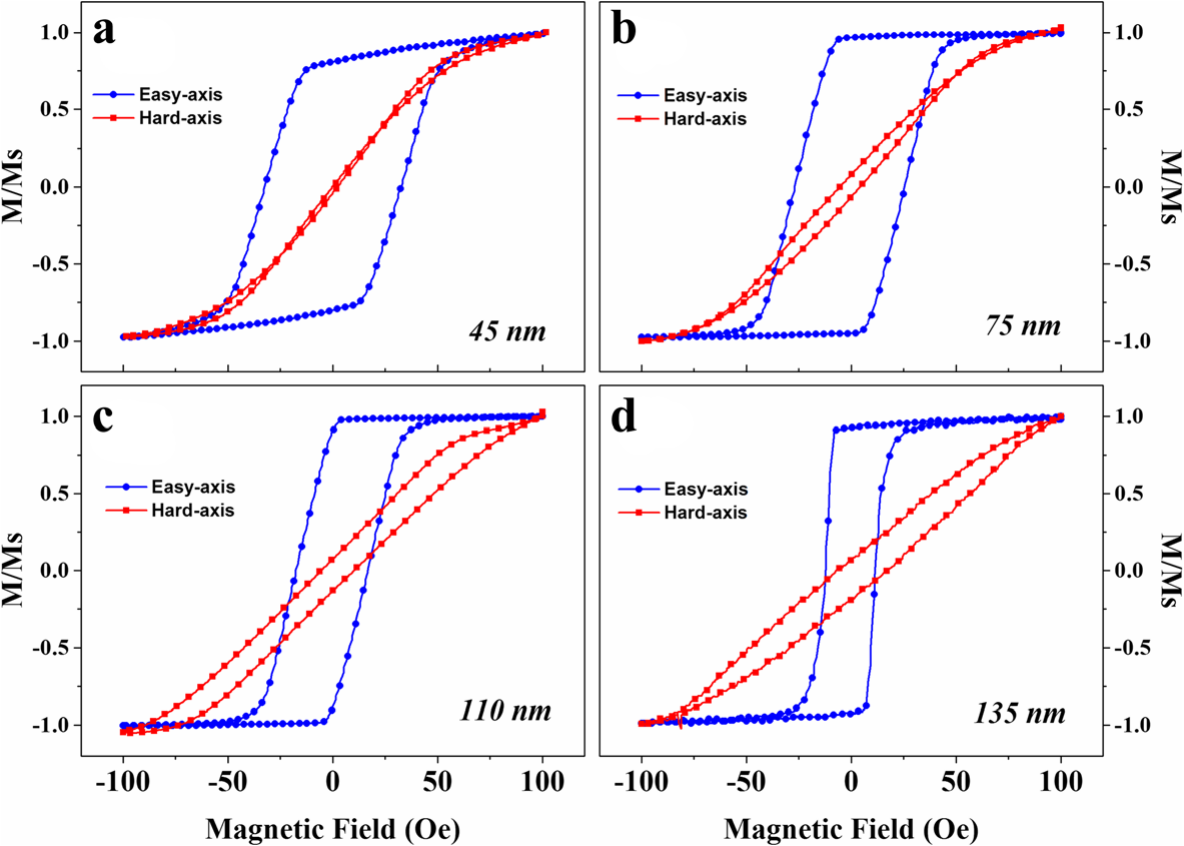
The hysteresis loops of combined stripe patterned magnetic films with different thickness. The results are exhibited from easy-hard axis defined by induced magnetic field direction in each picture. From **a** to **d**, the thickness of films varied from 45 to 135 nm

The static magnetic properties of combined stripe patterned films deposited at varying thickness from 45 to 135 nm were investigated. The easy axis was defined as the same as the direction of induced magnetic field whiles the hard axis was orthogonal to it, Fig. 2. Present parts of *M/Ms*-H loops of the films, which were measured at the field, range between 100 and − 100 *Oe*. The differences between easy axis and hard axis clearly show the induced in-plane uniaxial anisotropy, which was contributed by induced magnetic field as well as the stripe-shape-induced anisotropy. Furthermore, the hysteresis loops in Fig. 2 revealed well soft magnetic properties with *H*_*ch*_ as low as 13 Oe, where *H*_*ch*_ is the coercivity along the hard axis and *H*_*ce*_ is the coercivity along the easy axis. With increase of film thickness, *H*_*ch*_ would decrease from 32 Oe at 45 nm to 13 Oe at 135 nm, which was in accordance with random anisotropy model proposed by Herzer [19]. All details can be found in our former work [18].

**Fig. 3:**
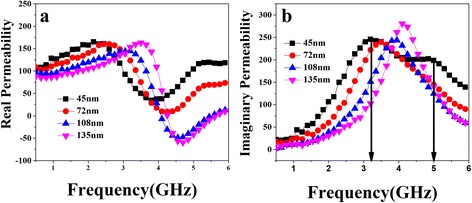
Permeability spectra measured at room temperature of combined stripe patterned FeCoBSi thin films with various thickness reveal the real permeability of films (**a**) and exhibit the imaginary permeability (**b**)

Figure 3 shows the real and imaginary components of the permeability spectra of combined stripe patterned films on the function of frequency with different thickness. It is interesting to find that for *t* = 45 nm, there are splitting resonance peaks that appear at *f*_low_ and *f*_High_ frequency over the measured frequency range, respectively. According to this graph, when *t* = 45 nm, the μ′ is high at about 170 while the *f*_low_ just reaches about 3.2 GHz and *f*_High_ is about 5 GHz. As the thickness increases, the value of *f*_low_ increases all the time. For *t* = 135 nm, we find that *μ′* can still remain at a proper level of 170, the *f*_low_ increases to a considerable value of 4.2 GHz simultaneously, while the *f*_*High*_ possibly is out of the measured frequency range 6 GHz. The resonance band, defined as full width half maximum (FWHM), was broaden to more than 4 GHz at thickness of 45 nm which is wider than the FWHM of double-stripe patterned films with 2 GHz [18]. It may pave a better way for future application as wide-band microwave EMI absorbers. The broaden band phenomenon was due to the different shape anisotropic field induced by five different width stripes. Consider the fixed width of the gap as 5 μm, which is large enough to magnetically separate the two consecutive stripes without coupling effect. Thus, each stripe was actually independent to each other leading to separate magnetic response under microwave excitation. The total response to the high frequency electromagnetic field should be a mathematical addition of five different width stripes. In addition, the shape anisotropy could play an essential role to determine the film’s effective anisotropy, i.e., resonance frequency [20]. Therefore, it is necessary to take account of demagnetization factor during micro-magnetic analyzation. In order to demonstrate the dynamic properties of our thin films, the LLG Gilbert equation formula [21] combined with demagnetization effect was used to describe the high frequency phenomenon for magnetic thin films with uniaxial anisotropy. Thus, the high-frequency permeability could be described by the following equation:

1 $$ \mu =1+\frac{2}{3}\frac{\gamma 4\pi {M}_s\left\{\gamma \left[{H}_e+4\pi {M}_s\left({N}_x-{N}_z\right)\right]+ i\omega \alpha \right\}}{\left\{\gamma \left[{H}_e+4\pi {M}_s\left({N}_x-{N}_z\right)\right]+ i\omega \alpha \right\}\left\{\gamma \left[{H}_e+4\pi {M}_s\left({N}_y-{N}_z\right)\right]+ i\omega \alpha \right\}-{\omega}^2} $$

where 4π*M*_*s*_ is defined as saturation magnetization, *α* is damping factor, *γ* is gyromagnetic ratio (1.76 × 10^7^*Oe*^−1^*s*^−1^ for FeCo alloy), *H*_*e*_ is effective anisotropy filed, and *N*_*x*_, *N*_*y*_, *N*_*z*_ is the demagnetization factor along three orthogonal directions, respectively. *f*_*r*_ can be derived by the Kittle equation as

2 $$ fr=\frac{\gamma }{2\pi }{\left\{\frac{\left[{H}_e+4\pi {M}_s\left({N}_y-{N}_z\right)\right]\left[{H}_e+4\pi {M}_s\left({N}_x-{N}_z\right)\right]}{1+2{a}^2}\right\}}^{1/2} $$

In light of stripes with different width included in our films, which induced distinctive shape anisotropy leading to split resonance peaks, the entire spectrum should be characterized as mathematical addition of five separate one. The demagnetization factor along *x*, *y*, and *z* direction can be written as [20]

3 $$ {N}_y=\frac{2}{\pi }{\tan}^{-1}\frac{T\sqrt{W^2+{T}^2+{L}^2}}{WL} $$

4 $$ {N}_x=\frac{2}{\pi }{\tan}^{-1}\frac{W\sqrt{W^2+{T}^2+{L}^2}}{TL} $$

5 $$ {N}_z=1-{N}_x-{N}_y $$

where *L* is the length along *z*-axis, *W* is the width along *x*-axis, and *T* is thickness along *y*-axis. With formula (3), (4), (5), and the LLG formula, the resonant frequency corresponding to different width of the magnetic stripe from 5 to 25 μm can be calculated, respectively.

**Fig. 4:**
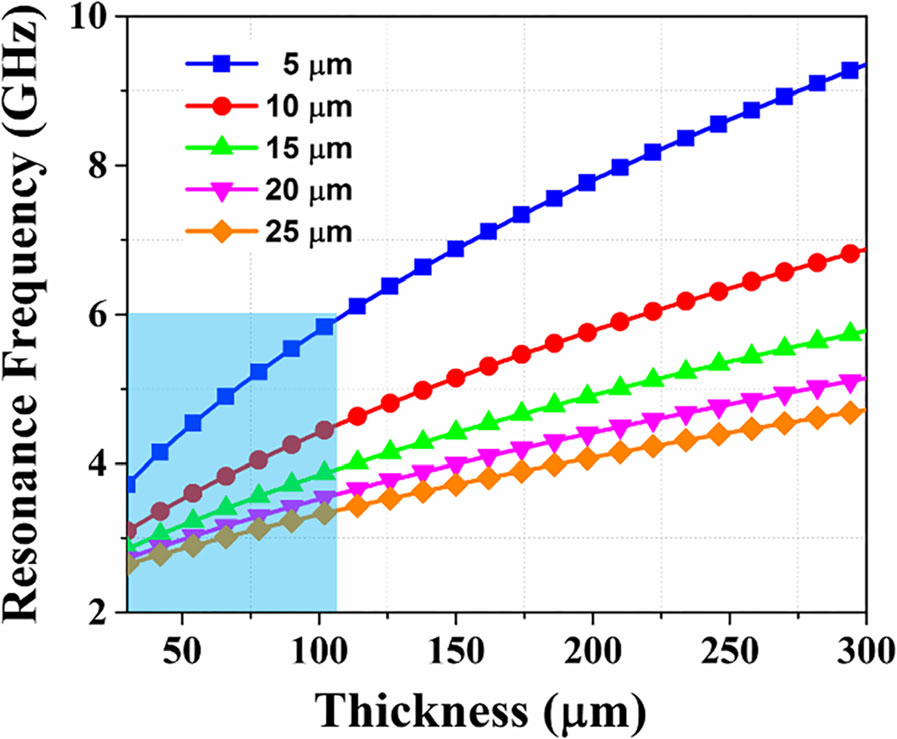
Numerical calculation of resonance frequency of different stripe width dependent on different thickness. The blue area reveals the available measurement frequency range (up to 6 GHz) for our setup

Figure 4 exhibits the calculated resonance frequency of distinct stripes with different thickness from 5 to 25 μm. In this calculation, *α* was set as 0.03, which had a little impact on the position of the resonance frequency. The saturation magnetization and effective in-plain anisotropy field, which were both extracted from the experimental results of continued FeCoBSi films, were set as 1345 emu/cm^3^ and 40 Oe [18], respectively. In the amorphous magnetic films, magnetocrystalline anisotropy could be ignored leading to a more essential role possessed by shape anisotropy in the resonance frequency determination process, which was demonstrated in [20]. Therefore, different width stripes should contribute to distinctive resonance peaks due to the decoupling effect maintained by the gap, results in multiple resonance peaks in the spectrum theoretically. Additionally, with the increase of film thickness, main resonance frequency would increase and the frequency difference between the stripes of different width (depicted in Fig. 4) was enhanced. Therefore, there is a strong super positioning effect between multiple resonance peaks if the thickness of film is thin enough in which case the band of magnetic spectrum showed a pronounced broadened behavior. With the increase of thickness, such superpositioning effect was weakened because of the more distinguish resonance frequency difference. With the thickness increased above 110 nm, the resonance frequency of stripes with certain width such as 5 μm was out of our measurement range as the blue area exhibited, resulted in a smaller FWHM compared to the 45 nm film. The resonance frequency could be predicted as well regarding of mathematical calculation. By tuning the width of stripes as well as the thickness of films, each resonance phenomenon could be controlled for actual application.

**Fig. 5:**
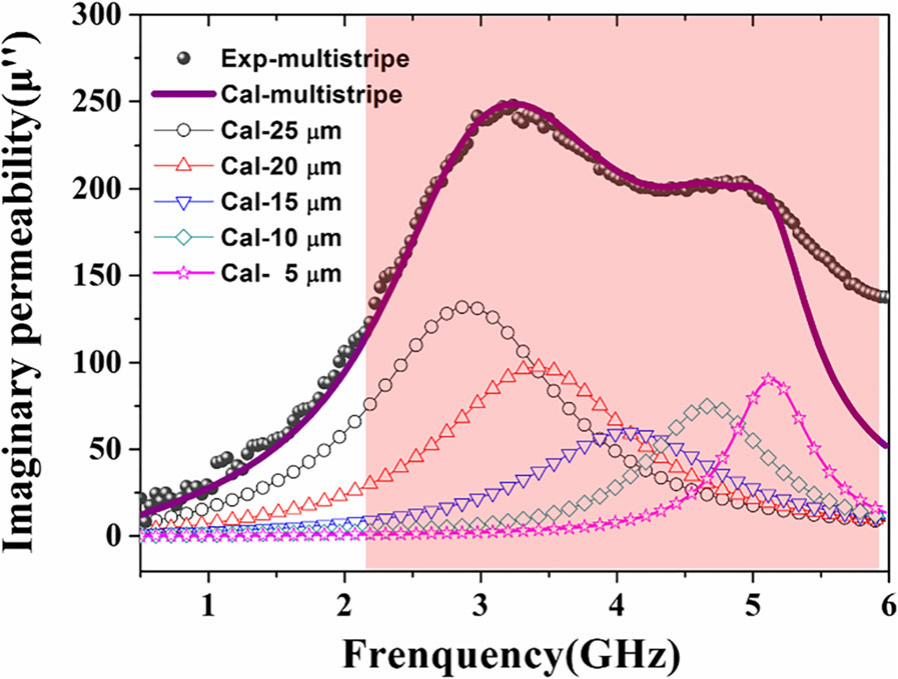
Measured and calculated imaginary permeability for combined stripe patterned FeCoBSi thin film with *T* = 45 nm and calculated imaginary permeability for stripes with different width. The red area corresponds to the resonance band (FWHM) of combined stripe patterned films

The assumption that the broaden band effect is due to the superposition of independent resonance peak induced by separate stripe can be understood clearly from the fitting result in Fig. 5. In order to verify our assumption, the magnetic spectrum of single-stripe patterned film was calculated as well. Compared to the combined stripe patterned film, the resonance frequency of each stripe fell in the range of FWHM of combined stripe patterned one as the red area depicted, which well supported our assumption that the broaden band phenomenon of combined stripe patterned film was due to superposition of distinctive resonance peaks induced by different stripes.

## Conclusions

In conclusion, we have studied the magnetic and microwave resonance characterization of combined stripe patterned FeCoBSi with different thickness. Compared to the former double-striped patterned films, five-striped patterned FeCoBSi pattern could extend the resonance band (FWHM) furthermore to 4 GHz. The broaden band phenomenon could be controlled by tuning width of different stripe as well as thickness of magnetic films in order to meet the requirement in the actual application, which may be useful in the future EMI devices.

## Abbreviations

EMI: Electromagnetic inference
FWHM: Full width half maximum
LLG: Landau-Lifshitz-Gilbert
